# Quality of Life, Physical Activity, and Mental and Physical Health Status in Croatian Middle-Aged and Elderly Population

**DOI:** 10.3390/healthcare13222931

**Published:** 2025-11-16

**Authors:** Manuela Maltarić, Mirela Kolak, Darija Vranešić Bender, Jasenka Gajdoš Kljusurić, Branko Kolarić

**Affiliations:** 1 Andrija Štampar Teaching Institute of Public Health, Mirogojska 16, 10000 Zagreb, Croatia; branko.kolaric@stampar.hr; 2 School of Medicine, University of Zagreb, Šalata 3, 10000 Zagreb, Croatia; mirela.kolak@student.mef.hr; 3 Faculty of Food Technology and Biotechnology, University of Zagreb, Pierottijeva 6, 10000 Zagreb, Croatia; dvranesi@kbc-zagreb.hr; 4 Unit of Clinical Nutrition, Department of Internal Medicine, University Hospital Centre Zagreb, Kišpatićeva 12, 10000 Zagreb, Croatia

**Keywords:** gerontology, dietary habits, obesity, chronic diseases, public health, elderly

## Abstract

**Background/Objectives**: The proportion of middle-aged and elderly people in the total population is increasing, and it is of utmost importance to monitor their quality of life (QoL), which largely depends on mobility, health and mental state, diet, nutritional status (especially overweight and obesity). The population in Croatia is among the leading in terms of the proportion of overweight and obese people, therefore the aim is to study QoL and determine which aspects can potentially be mitigated by public health actions. **Methods**: In accordance with the available data from the SHARE study (Survey on Health, Aging and Retirement in Europe), data were taken from the most recently published—9th wave conducted in 2021/2022. In this study, the Croatian population older than 50 years is represented by 4687 respondents. Health-related parameters were monitored (cardiovascular diseases, diabetes, mental health, handgrip strength (HGS) as a biomarker in older people and body mass index) and quality of life (self-assessed quality of life (CASP, self-assessed health SPH, physical activity) and dietary habits. A logistic regression model was used to link HGS as a biomarker in older people with quality of life and health parameters. **Results**: There is an undeniable decline in social and physical activity with age; the proportion of people engaged in vigorous physical activity decreased from 47% in the 51–64 age group to only 5.4% in people over 85 years of age, while physical inactivity increased from 3% to 37.7%. Chronic diseases, especially hypertension, accumulate with age, while self-rated health worsens with age, as does mental health (the proportion of depressed people (according to the EURO-D scale) increased significantly from 21.1% in the 51–64 age group to 54.1% in those over 85 years of age). Results of multinomial logistic regression showed that sports (in)activity was consistently associated with a lower likelihood of reduced handgrip strength (OR = 1.94 for low strength, *p* < 0.001). **Conclusions**: Sports activities and social engagement are crucial for maintaining good handgrip strength. Higher BMI, lower education and adverse psychological states are risk factors for a weaker handgrip. These findings highlight the need for an integrated public health approach that promotes physical activity, balanced nutrition and mental and social well-being in the older population.

## 1. Introduction

### 1.1. Prevalence and Impact of Obesity and Chronic Disease in Croatia

Data from the European Health Interview Survey (EHIS) conducted in Croatia show that just 33.9% of adults aged 18 and over fall within the normal BMI range (18.5–24.9 kg/m^2^). In contrast, 64.8% of adults are considered either overweight or obese, and 1.4% are classified as undernourished. Croatia, along with Malta, ranks at the top among EU nations for the highest percentage of adults with excess weight. When looking at the data by gender, Croatia has the highest rate of overweight or obese individuals in the EU for both sexes—73.2% of men and 58.5% of women fall into these categories. Obesity specifically affects 23.0% of Croatian adults, with a slightly higher prevalence in men (23.7%) compared to women (22.6%). For older adults in Croatia, the 2019 EHIS reports that 25.4% have a healthy body weight. Meanwhile, 45.7% are overweight and 28.4% are obese. Only 0.5% of this group are undernourished. Among this demographic, normal weight was found in 24.2% of men and 26.1% of women. However, more men were overweight (51.4%) than women (41.9%), while obesity was more frequent among women (31.2%) compared to men (24.4%) [1,2]. According to the World Health Organization, obesity refers to an excessive accumulation of body fat that poses a risk to health. It is commonly identified when an individual’s body mass index (BMI) exceeds 30 kg/m^2^ [3]. Obesity has widespread effects on nearly all organ systems and significantly increases the risk of various serious health conditions. The most notable among these are type 2 diabetes (T2DM), high blood pressure (hypertension), abnormal cholesterol levels (dyslipidaemia), heart disease (cardiovascular disease), and certain types of cancer [4]. Excess body weight has been shown to directly shorten life expectancy [5,6]. The occurrence of obesity is influenced by both inherited genetic traits and external environmental factors [7]. Nonetheless, obesity caused by mutations in a single gene—known as monogenic obesity—is uncommon, accounting for fewer than 1% of all cases [8]. Large-scale genome-wide association studies have uncovered many genetic variants linked to obesity traits such as BMI, appetite, energy balance, fat metabolism, and brain pathways controlling eating, yet these genes account for only around 5% of individual differences in obesity [9,10,11,12]. The heritability of obesity is also shaped by epigenetic mechanisms—reversible changes to DNA that influence gene activity without altering the sequence itself—with research showing that factors like diet, physical activity, weight changes, and bariatric surgery can lead to tissue-specific shifts in DNA methylation patterns [9,13]. However, studies indicate that these epigenetic modifications are mostly a result of increased body fat rather than a cause, and the underlying mechanisms driving these changes remain largely unclear [14]. The most significant health conditions associated with obesity are cardiovascular disease, type 2 diabetes, cancer, cardiovascular disease and diabetes accounting for the majority of related healthcare expenses [15,16,17,18]. Additionally, obesity causes changes in fat tissue structure and reduced oxygen supply (hypoxia), further contributing to these inflammatory and metabolic disturbances [19]. Research has also found that fat tissue in obese individuals secretes higher levels of inflammatory molecules such as IL-6 and TNF-α, which are associated with insulin resistance [20]. Obesity is characterized by immune changes. Free fatty acids and their intermediates can activate toll-like receptor 4, leading to the recruitment of immune cells. In adipose tissue from individuals with obesity, both innate and adaptive immune responses are evident, with increased infiltration and activation of macrophages and T-cells [21,22,23,24,25]. Obesity induces widespread alterations in insulin signalling, especially in the liver and skeletal muscle, which play key roles in regulating glucose balance. Research indicates that higher concentrations of free fatty acid intermediates—particularly diacylglycerol—in the liver are linked to insulin resistance [26]. The inflammatory and metabolic alterations described earlier happen both locally and throughout the body, leading to considerable damage to the cardiovascular system. In obesity and dyslipidaemia, elevated lipoproteins such as LDL and chylomicrons can penetrate the vascular sub-endothelium, triggering an immune-driven inflammatory response that ultimately results in atherosclerosis [27]. Lipid particles trapped in the arterial intima can undergo oxidation, which promotes activation of endothelial cells, increases the expression of adhesion molecules, and leads to the build-up of leukocytes beneath the endothelium [28]. Macrophages filled with lipids, called foam cells, drive local tissue damage and cell death, leading to the accumulation of chemokines and antigens that can attract T-cells and promote the development of atherosclerotic plaques [29].

### 1.2. Importance of the Lifestyle Factors and Functional Measures Like Handgrip Strength

Handgrip strength (HGS) is an important indicator of health status in older adults, and the literature highlights its importance as a biomarker that is associated with physical activity, overweight, and general health [30,31,32,33,34]. HGS is used to assess muscle strength, because it is based on neuromuscular function and is a reliable measure of neuromuscular integrity and muscle function [31]. The measurement is performed with a dynamometer and the strength of the dominant hand and/or both [32] can be measured, and the low limit values of HGS are taken as values < 27 kg for men and <16 kg for women [30].

HGS is a key biomarker of health in older age due to its association with negative health outcomes and represents a simple, rapid, and inexpensive way to stratify an individual’s risk of comorbidity and mortality [31,32,33]. It also represents a prognostic indicator of future adverse conditions (disability and adverse outcomes, such as prolonged hospitalization and increased risk of complications after hospitalization or surgery). Low BMI is associated with an increased risk of all-cause and cardiovascular mortality and is fundamental to the diagnosis of frailty and sarcopenia. Sarcopenia is a syndrome defined by age-related decline in skeletal muscle mass and function. When considering the association of BMI with physical activity, body weight (obesity) and health, key recommendations from the World Health Organization (WHO) are at least 150 min of moderate-intensity or 75 min of vigorous-intensity physical activity per week, which is associated with a reduced prevalence of low BMI. The study of Doyev et al. [30] showed that subjects who engaged in physical activity were more likely to have normal BMI while reduced levels of physical activity were significantly associated with lower BMI. The association of HGS with body mass index (BMI) is inconsistent, as BMI is not an indicator of muscle strength (it does not reflect body composition), which is particularly relevant for older adults [30,31].

When considering the association of HGS with general health and nutrition, the prevalence of low HGS increases significantly with age. Higher education is associated with a reduced prevalence of low HGS, while comorbidities (chronic diseases, NCDs) are more common in the group with low HGS [31]. Low HGS is a predictor of mortality from specific causes, including death from cardiovascular diseases (CVDs) and death from cancer [32]. Research on HGS and dietary patterns shows that an increase in energy intake by 100 kcal/day reduces the prevalence of low HGS by approximately 5% [30]. Low energy intake is associated with lower muscle strength, which can be explained by the fact that lack of energy leads to muscle catabolism for energy [30]. It should also be emphasized that asymmetry of the hand grip, i.e., a strength ratio of the non-dominant/dominant hand ≥ 20% may reflect abnormal function of the nervous system and neuro-motor system, which increases the risk of falls [32]. Therefore, it is undeniable that it is necessary to monitor HFS in the elderly population as an indicator of general health status.

### 1.3. Study Rationale and Objectives

The SHARE study provides insight into data on quality of life parameters, physical activity, self-perception of health status and life satisfaction, as well as health indicators (physical and mental), but also biomarkers such as hand grip strength. Given that in Croatia a significant proportion of the population over 50 years of age is 43.72% (21.38% aged 50–65 and 22.34% over 65), it is necessary to monitor their quality of life, health and nutritional status in order to determine critical factors that influence their QoL, regardless their age.

In accordance with all previously mentioned, it is evident that, in the population over 50 years of age, it is of critical importance to monitor the quality of nutrition, physical activity, as well as the mental and physical status. Therefore, the aim of the study was to investigate the relationship of HGS, a biomarker in older people, with available health outcomes, self-rated quality of life (CASP-12) and health (SPH) parameters, diseases (with special emphasis on CVDs and diabetes), dietary habits, mental health indicator (EURO-D), physical activity and body mass index.

## 2. Materials and Methods

### 2.1. Data

For the purposes of this study, data from the SHARE project (Survey on Health, Ageing and Retirement in Europe), wave 9 (2021/22), [30] were used. The SHARE project has been collecting microdata on health, socioeconomic status, and social and family networks for the European population aged 50 and over since 2004, and Croatia has been participating since wave 6 [17]. Data collection is unified in all countries and is conducted through interviews (face-to-face) with computer-assisted personal interviewing (CAPI). For wave 9, data were collected from respondents (*n* = 69,447) from Austria, Germany, Sweden, the Netherlands, Spain, Italy, France, Denmark, Greece, Switzerland, Belgium, Israel, the Czech Republic, Poland, Luxembourg, Hungary, Slovenia, Estonia, Croatia, Lithuania, Bulgaria, Cyprus, Finland, Latvia, Malta, Romania and Slovakia). But for the purpose of this study, data for the Croatian population were excluded (n = 4687, which makes up 6.7% of the European population covered in wave 9 of the SHARE study).

### 2.2. Variables

**Figure 1:**
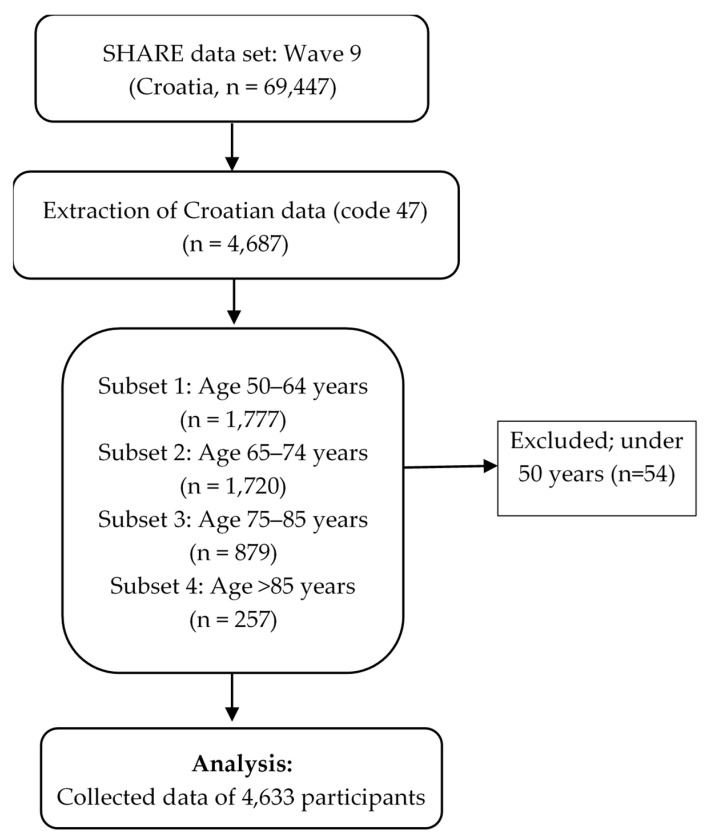
Flowchart of the data inclusion.

The basic indicators of the included population group are presented in Table 1, and the data analysis workflow is shown in the flowchart (Figure 1).

In the dataset, with the aim of providing a clearer insight into changes in the quality of life, activities, health and habits of the Croatian population over 50 years of age, data from the SHARE study for wave 9 were extracted according to the flowchart in Figure 1.

#### Health-Related Indicators

As indicators of population health, (i) the indicator of chronic diseases related to cardiovascular diseases (myocardial infarction and other heart problems, hypertension, high blood cholesterol, stroke) and type 2 diabetes; (ii) physical activity level; (iii) handgrip strength (HGS); (iv) control, autonomy, self-realization, and pleasure scale (CASP-12) and the (v) self-perceived health (SPH) [30].

The CASP-12 index is one of the most common international measures used for assessment of quality of life (QoL) in older adults [31]. To assess the CASP-12 index, the questions include a list of statements used to describe the respondent’s life and how they feel and think, with responses coded on a scale indicating how often, if at all, the respondent experienced these feelings and thoughts. The scale uses 4 levels from “Often”, through “Sometimes” and “Rarely” to “Never”. The CASP-12 index ranges from 12 to 48. To indicate the measure of depressive symptoms in elderly, the EURO-D parameter was used. This parameter includes twelve symptoms commonly associated with depression: (i) Depression; (ii) Pessimism; (iii) Suicidality (“life not worth living”); (iv) Guilt; (v) Sleep problems; (vi) Loss of interest; (vii) Irritability; (viii) Appetite changes; (ix) Fatigue; (x) Lack of concentration; (xi) Lack of enjoyment and (xii) Tearfulness. Each of mentioned symptom is scored (0 = absent or 1 = present), so the final score can range from 0 to 12. Score ≥ 4 is used as an indicator of clinically relevant depression [31].

A biomarker that indicates maintained physical health is the maximum static force of the hand grip and is measured with a dynamometer. It’s a method that’s simple and cheap, and its Max Grip that’s linked to for evaluation. increased risk of chronic diseases and multimorbidity and mortality, in both sexes [19,22]. Low handgrip strength was defined by Doyev et al. as values < 27 kg for men and <16 kg for women [30], and the medium HGS group was set from 27–40 kg for men and 16–24 kg for women, while values above the mentioned ones are considered as high HGS.

### 2.3. Data Analysis

Descriptive statistics were used to describe the basic characteristics of the participants. Categorical variables were presented as frequencies and percentages, while numerical variables were presented as mean and standard deviation (or median and interquartile range, depending on the distribution). Differences between groups in categorical variables were examined using the chi-square test. Participant characteristics for continuous variables are expressed as means and standard deviations and as frequencies (n) or percentages (%) for categorical variables. To determine statistically significant differences between groups, one-way ANOVA with Tukey HSD post-hoc when assumptions held were used (with a significance level of *p* < 0.05). Multinomial logistic regression analyses were also performed to analyze the associations of the three categories of handgrip strength with health parameters, activities, and general population indicators. Age, gender, gender, age, level of education, BMI and marital status were included as covariates to control for their potential confounding effect on the relationship between HGS and the observed variables of health status, self-rated health and quality of life parameters, and physical activity. Relative risks were estimated using odds ratios (ORs) and 95% confidence intervals (CIs). All statistical analyses were performed using IBM SPSS (version 19; IBM, Armonk, NY, USA).

## 3. Results

Cross-tabulations were used to monitor (i) the trend in changes in the subjects’ physical activity, (ii) their changes in the frequency of consumption of certain types of food, by age group, and (iii) changes in their physical and mental health. The participation of subjects in leisure activities and physical activities by age group is shown in Table 2. The results show a clear decline in social and physical engagement associated with aging.

Results indicate that within the Croatian middle-aged and elderly population, volunteering and learning decline sharply with age, with volunteer work falling from 6.3% (aged 51–64) to only 0.4% (>85). Club attendance and activities in them decline with age (12.3% → 1.2%), as does political/community involvement (2.7% → 0.4%). Reading is common across all groups but has a declining trend with age (50.3% → 28.0%), while word/number games peak slightly between the ages of 65 and 74, but then decline as does playing cards/chess (19.2% → 3.7%). Observation of the physical activity, vigorous activity declines sharply with age. In the 51–64 age group, 47% exercise vigorously more than once a week, compared to only 5.4% in the >85 age group. Accordingly, physical inactivity increases with age (never active: 3% → 37.7%).

The potential change in dietary patterns that can be monitored from the SHARE data is described by the frequency of consumption of (i) dairy products, (ii) legumes and eggs, (iii) meat, fish and poultry, and (iv) fruits and vegetables. Changes in the consumption of these four food groups, by age group, are listed in Table 3.

Dairy consumption is the only one that does not show statistically significant differences depending on the frequency of consumption in different age groups (Pearson Chi-square = 30.273; *p* = 0.1759). Daily consumption increases slightly with age (47.9% → 58.8%). Daily intake of legumes/eggs decreases with age (10.5% → 8.2%), while the intake of those who eat them less than once a week increases significantly (>85 years: 14.4%). Daily consumption of foods from the category Meat/fish/poultry decreases with age (54.3% → 46.3%), although older adults still maintain a moderate weekly intake. Daily consumption of fruits and vegetables is high in all age groups, and the statistically significant difference is due to the representation of those respondents who consume it once or even less times a week (Pearson Chi-square = 42.353; *p* = 0.0118). While consumption of fruits/vegetables and dairy products remains stable or improves, protein-rich foods such as meat/eggs are declining, especially in the oldest age group (85+).

In public health monitoring of the population, information on physical and mental health is of utmost importance, as is personal perception, and an overview is given in Table 4.

The results show that in Croatian middle-aged and older respondents, chronic diseases accumulate with age, and almost half of people over 85 have 3 or more diseases (49%). Cardiovascular problems and diabetes are positively correlated with age, especially hypertension. Hypertension increases from 38.1% in the age group 51–64, to 66.1% in the population over 75. Observing self-rated health (SPH), it worsens with increasing age of respondents, and only ~5% of people over 85 rate their health as very good/excellent.

Mental health was assessed according to the Depression scale EURO-D parameter [30], and there was a significant increase in respondents who were classified as depressed according to the mentioned parameter. At the age of 51–64, only 21.1% of respondents were classified as depressed, while a significant increase was seen in the age group 75–80 (36.8%) and especially in people over 85 (54.1%). The differences are significant (Pearson Chi-Square 288.948; *p* < 0.0001). The EURO-D parameter has 12 features that are assessed and they are also listed in Table 4, and it is clear that the mental state of the subjects also deteriorates with age. Thus, depression, fatigue, concentration problems and pessimism increase with age, and suicidality almost quadruples from middle age (3.6%) to late old age (12.5%).

**Figure 2:**
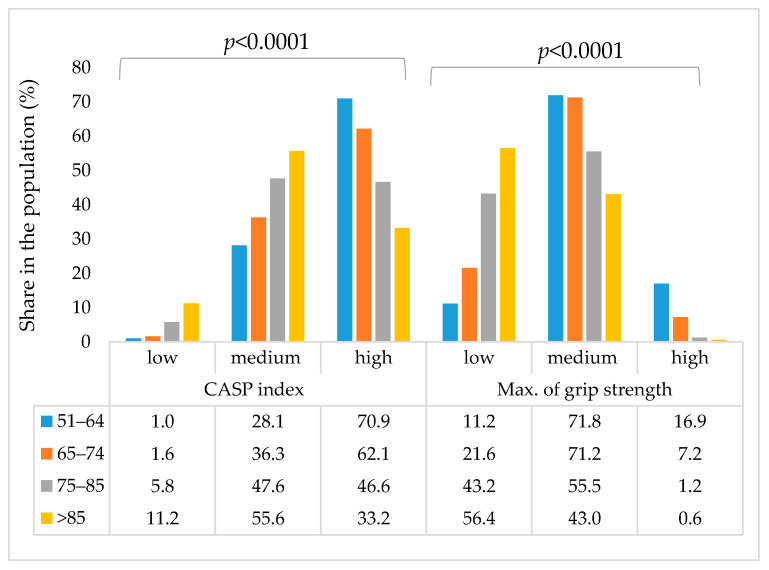
Changes in CASP-12 index and maximum handgrip strength (HGS) in Croatian middle-aged and elderly population, according to different age groups. (CASP-12: index of self-assessment of QoL and well-being).

In the SHARE study is also collected the measure of quality of life (QoL) in older ages, the Control, Autonomy, Self-realization, and Pleasure scale, CASP-12 [34,35], as well as the maximum Handgrip strength measure, which is proposed as a biomarker of the body function [30,31,32,33]. Therefore, the two parameters mentioned above are shown in Figure 2.

In Figure 2 are presented the distributions of two indicators related to the health of the middle-aged and elderly population (i) CASP-12 index and (ii) HGS, for different age groups (51–64, 65–74, 75–85 and >85 years).

Thus, a high CASP index (a measure of quality of life and well-being) is more common in younger groups (70.9% at the age of 51–64; 62.1% at the age of 65–74), and decreases with age (46.6% at the age of 75–85; 33.2% at the age of >85). The intermediate category for the CASP index increases with age (28.1% → 55.6%), which indicates that a larger proportion of respondents, with increasing age, “transitions” from the high category to the intermediate category as they age. A low CASP index is rare at a younger age (1.0%), but has a significant increase towards the oldest age group (11.2%). Subjective assessment of general well-being (CASP-12), i.e., CASP category, decreases significantly with increasing age, which is also confirmed by statistical indicators (Pearson Chi-Square 500.021; *p* < 0.0001).

On the other hand, maximum hand grip strength (a marker of physical function) shows that, as expected, high grip strength is relatively common in the youngest group (16.9%), but drops sharply with age, almost disappearing after 85 years (0.6%). Medium grip strength dominates in middle age (71.8% for 51–64; 71.2% for 65–74) and also decreases with age (55.5% → 43.0%), while low grip strength increases from 11.2% at age 51–64 to more than half (56.4%) at age > 85. The general trend of decreasing physical strength (maximal hand grip) significantly decreases with age (Pearson Chi-Square 970.635; *p* < 0.0001).

With the help of a multinomial logistic-regression model, observed adjusted odds ratios for all analyzed parameters of activity (free and physical activities), health (physical and mental), objective and subjective satisfaction, and nutrition (Table 5).

The model investigated the relationship of the dependent variable “Handgrip strength category” where the “high” category was taken as the reference and the groups “medium” and “low” were compared. The following independent variables were observed: activities (social, sports, volunteer), food intake, cardiovascular/metabolic conditions, EURO-D depression indicators, CASP quality of life, self-rated health (SPH), BMI, retirement status, marital status and level of education. The output parameters are presented: regression coefficients (B), odds ratios (OR with 95% CI) and *p*-values. Physical inactivity shows that not engaging in sports activities was consistently associated with a lower likelihood of reduced grip strength, with an OR of 1.45 (95% CI 1.23–1.70, *p* < 0.001) for medium strength and an OR of 1.94 (95% CI 1.59–2.37, *p* < 0.001) for low strength. The activities in which the middle-aged and older population participate have negative B-values (“done voluntary or charity work”; “attended an educational or training course”; “gone to a sport, social or other kind of club”; “taken part in a political or community-related organization” and “played cards or games such as chess”), indicating that social activity correlates with better grip strength.

When observing dietary habits, a significant association was observed in the low- strength group (OR = 0.78, CI 0.62–0.97, *p* = 0.024), which is probably a consequence of increasing protein-rich foods that can help preserve muscle strength.

Hypertension showed a borderline association with medium strength (OR = 1.52, *p* = 0.054), while other health conditions (heart attack, cholesterol, stroke, diabetes) did not show a significant association, while the number of chronic diseases was significantly associated with low handgrip strength (OR = 1.31, *p* = 0.058).

When observing the mental health parameter (EURO-D), irritability significantly increases with a decrease in handgrip strength (OR = 0.54, *p* = 0.048).

Quality of life parameters (CASP and SPH) are strong positive predictors, and with increasing CASP category (low, medium to high) the incidence of low grip strength category decreases (OR = 9.99, CI 1.08–92.77, *p* = 0.043). However, this parameter shows wide ranges in CI, which may be due to sample size or variability [35].

Looking at the basic sociodemographic factors, higher BMI increases the probability of lower grip strength (medium: OR = 0.55 (*p* = 0.017) and low: OR = 0.43 (*p* = 0.005)). Retirement did not show a significant association; however, single life (marital status) showed an association with weaker grip strength (OR = 1.39, *p* < 0.001), as did lower education, which increased the likelihood of a weaker grip (OR = 0.91, *p* = 0.021).

## 4. Discussion

In discussing the health and well-being of the population over 50 years of age, it is crucial to consider various interrelated factors such as physical activity, leisure activities, mental and physical health, dietary habits and hand grip strength. These factors not only influence the quality of life of older people, but also the risk of developing chronic diseases and functional decline. Quality of life (QoL) in older people shows a significant linear decline over time, although older people often report high QoL and life satisfaction. QoL is a multidimensional, subjective and measurable construct that encompasses physical and psychological state and social functioning. Negative emotions, especially stress and anxiety, strongly predict a decline in QoL, while depression did not show a significant effect in the study by Ma et al. [36]. High levels of negative emotions contribute to a lack of resources and adaptive capacity, which can result in a poorer QoL. Unmet health needs significantly affect QoL and exacerbate health inequalities. During the COVID-19 pandemic, older people in Europe faced barriers to accessing healthcare, including avoidance of treatment due to fear of infection, delayed medical appointments and denial of care [37]. In terms of cognitive health, dementia represents a significant global burden, with the number of sufferers projected to increase to 139 million by 2050 [38]. Risk factors for dementia include low education, hypertension, hearing impairment, smoking, obesity, depression, physical inactivity, lack of social engagement, excessive alcohol consumption, traumatic brain injury and air pollution. Depression negatively affects cognition, but higher sports social capital can reduce its detrimental impact [39]. Physical activity has been recognized as a low-cost and non-pharmacological intervention for the prevention of dementia, improving physical health and cognitive function by stimulating brain plasticity [38]. In addition, it promotes social interaction and strengthens social capital. The term “sport social capital” refers to the social resources built through sports activities, including trust, emotional support, social networks and a sense of belonging. This form of capital plays a unique role in maintaining cognitive function and enhancing cognitive reserve. Long-term participation in sports activities and the accumulation of social capital result in slower cognitive decline. It is important to emphasize that sports social capital is more effective in promoting cognitive health than physical activity alone. Participation in sports activities provides a platform for older adults to reconnect with peers, create new social networks and alleviate feelings of loneliness, improving mental resilience and cognitive health. Promoting group sports activities that foster social support networks may be an effective intervention, especially for groups with lower education [40].

Low physical activity is a risk factor for ischemic stroke [41], while regular exercise (strength training, aerobic, yoga, or Pilates—300 min/week moderate or 150 min/week vigorous) helps reduce visceral fat and improves handgrip strength (HGS), lowering the likelihood of low HGS by 47% [42]. Aging often leads to reduced physical and social activity, contributing to physical decline and mental health challenges, while diet quality generally remains adequate, though protein underconsumption is a risk.

Both subjective quality of life (CASP index) and physical ability (HGS) decline with age, with younger adults clustering in “high” categories and older adults in “medium” or “low” categories. HGS is a key marker of overall muscle strength and health in older adults, associated with physical function, fall risk, quality of life, disability, and mortality [43,44]. Age, education, and sex influence HGS—older age and lower education increase risk of low HGS, while men generally have higher HGS. Energy intake, especially 100 kcal/day increases, supports muscle strength and bone health, with chewing ability particularly relevant in those aged 65–74 [30,40]. Oral health affects dietary intake, as chewing difficulties can lead to avoidance of certain foods, negatively impacting nutrition and muscle strength [45,46,47]. High energy intake correlates more strongly with HGS than individual macronutrients.

Obesity increases risks of cardiovascular disease (CVD), type 2 diabetes, osteoarthritis, mobility limitations, and respiratory problems. Higher BMI correlates with higher CVD prevalence, stroke, hypertension, and diabetes [43]. For adults over 65, BMI should follow ESPEN guidelines: <18.5 severe underweight, 18.5–20.9 underweight, 21–27.4 normal, 27.5–30.9 overweight, 31–39.9 suspended, ≥40 high risk [47].

Chronic diseases and mental health issues become more common with age, reducing self-rated health [48]. Key determinants of strength in Croatians aged 50+ include physical activity, diet quality (legumes/eggs), mental health, BMI, marital status, and education. Sports participation and social engagement help maintain HGS, while higher BMI, lower education, and adverse psychological states increase risk of weaker grip.

However, the strengths and weaknesses of this research should be emphasized. The strengths of the study are certainly international comparability, as the SHARE study [49] is a multinational study that allows for comparisons between Croatia and other countries, which is useful in shaping policies and understanding the broader context of population aging (planning health, pension and social policies). The SHARE study collects data through multiple waves, thus enabling monitoring of changes over time and finding cause-and-effect relationships. The study has a multidisciplinary approach by encompassing health, economic, social and family aspects of the lives of people over 50 years of age, which allows for comprehensive analyses. The study uses the results of standardized questionnaires and data controls, which ensures reliability. However, a weakness should be highlighted as the sample size for more detailed sub-analyses, e.g., regional differences according to NUTS-2 regions (which in Croatia would be: Pannonian Croatia, the city of Zagreb, Northern Croatia and Adriatic Croatia). Part of the data is based on self-assessment by respondents, which may lead to inaccurate results. But each new wave of data collection seeks to resolve potential ambiguities, and so in wave 9 the question of reasons for not eating meat was introduced for the first time, as the number of vegetarian dietary patterns has begun to grow significantly in recent years [49].

Regarding specific dietary habits in Croatia, research shows that the Croatian population, especially men, consume significantly more meat, fish or chicken on a daily basis compared to the EU average. This habit may be associated with an increased risk of CVD.

Common cardiovascular/metabolic diagnoses (diabetes, stroke, cholesterol) show a limited direct association, while quality of life measures (CASP) show a potential impact, although with unstable estimates. The financial inaccessibility of meat is a significant problem in Croatia, especially for the middle-aged population and those with malnutrition or normal BMI. Although the patterns of consumption of dairy products and legumes/eggs are similar in the EU and Croatia, there are statistically significant differences in the daily consumption of dairy products in the older population and in the consumption of legumes/eggs and fruits/vegetables 3–6 times a week in the older population [48]. A diet low in fruits and vegetables, alcohol consumption and high BMI are risk factors for stroke. Mediterranean and ketogenic diets and high-protein diets can reduce visceral fat and preserve muscle mass.

## 5. Conclusions

The high dependence of QoL and HGS on lifestyle and mental health factors highlights the urgent need for a public policy focused on promoting active aging in Croatia. Engagement in sports and social activities emerged as particularly important for maintaining optimal handgrip strength. Multinomial logistic regression indicated that no participation in sports was consistently associated with a lower likelihood of reduced handgrip strength, with an odds ratio (OR) of 1.94 for low handgrip strength (*p* < 0.001). Similarly, involvement in social activities, including attending clubs or volunteering, was associated with higher handgrip strength. Individual chronic conditions such as cardiovascular disease and diabetes were not directly associated with handgrip strength in the regression model, but the cumulative number of chronic diseases showed a marginally significant association with low handgrip strength (OR = 1.31, *p* = 0.058). These findings underscore the importance of a comprehensive public health strategy that promotes regular physical activity, balanced nutrition with adequate protein intake, and the maintenance of mental and social well-being in older adults. Handgrip strength remains a reliable indicator of overall muscular health and a predictor of functional status, disability, and mortality. Sustaining participation in sports and social activities may help slow cognitive decline and preserve handgrip strength, serving as a valuable biomarker of health and functional resilience in middle-aged and elderly populations.

## Figures and Tables

**Table 1:** Basic characteristics of the population included in the study.

Variable	Frequency *n* (%)
Gender	
Male	2040 (43.5)
Female	2647 (56.5)
Age (years)	
50–64	1777 (37.9)
65–74	1720 (36.7)
75–85	879 (18.8)
>85	257 (5.5)
Living with partner	1677 (35.8)
Retired	2994 (63.4)
Normal Body mass index (kg/m^2^)	1280 (27.3)

**Table 2:** Respondents’ participation in various leisure and physical activities, depending on age group.

Leisure and Sport Activities	Age Group				*p*-Value *
	51–64	65–74	75–85	>85	
Activities in the last year:					
done voluntary or charity work	6.3	3.6	1.7	0.4	<0.0001
attended an educational or training course	4.4	0.9	0.2	0.0	<0.0001
gone to a sport, social or other kind of club	12.3	10.1	4.8	1.2	<0.0001
taken part in a political or community-related organization	2.7	2.6	1.7	0.4	<0.0001
read books, magazines or newspapers	50.3	49.5	42.7	28.0	<0.0001
did word or number games (crossword puzzles /Sudoku./etc.)	24.2	24.9	20.3	12.6	<0.0001
played cards or games such as chess	19.2	15.7	8.3	3.7	<0.0001
Sports or activities that are vigorous					
More than once a week	47.0	30.6	17.1	5.4	<0.0001
Once a week	14.6	14.0	12.3	3.9	
One to three times a month	14.7	16.3	12.9	5.4	
Hardly ever, or never	23.4	39.0	57.1	84.8	
Physical inactivity					
Other	96.6	93.3	81.8	61.9	<0.0001
Never vigorous nor moderate physical activity	3.0	6.5	17.4	37.7	

* The χ^2^ test was used to compare categorical variables.

**Table 3:** Eating habits of respondents by age group.

Frequency of Consumption	Age Group				*p*-Value *
	51–64	65–74	75–85	>85	
Dairy products					
Every day	47.9	49.4	51.3	58.8	0.1759
3–6 times a week	26.1	25.6	25.1	23.7	
Twice a week	12.1	12.9	13.2	8.9	
Once a week	5.8	5.4	4.8	4.3	
Less than once a week	7.6	6.5	4.8	3.9	
Legumes or eggs					
Every day	10.5	10.5	8.8	8.2	<0.0001
3–6 times a week	30.2	32.2	31.2	30.7	
Twice a week	36.6	34.3	34.2	29.6	
Once a week	18.0	18.6	17.9	16.7	
Less than once a week	4.2	4.1	7.2	14.4	
Meat, fish or poultry					
Every day	54.3	52.6	44.4	46.3	0.0018
3–6 times a week	37.5	39.0	44.0	42.0	
Twice a week	5.6	6.1	7.4	7.0	
Once a week	1.5	1.5	1.6	3.1	
Less than once a week	0.5	0.6	1.7	1.2	
Fruits or vegetables					
Every day	78.8	79.1	76.0	81.3	0.0118
3–6 times a week	14.3	15.5	15.6	13.6	
Twice a week	4.0	3.1	5.3	3.9	
Once a week	2.1	1.5	0.8	0.8	
Less than once a week	0.3	0.5	1.5	0.0	

For each food category, there were respondents who did not provide an answer, and accordingly, within a certain age group, the sum of the shares may be less than 100%. * The χ^2^ test was used to compare categorical variables.

**Table 4:** Physical and mental health by age groups.

Health Parameter	Age Group				*p*-Value *
	51–64	65–74	75–85	>85	
Number of chronic diseases					
0	31.6	16.4	7.2	7.4	<0.0001
1	28.1	25.1	21.3	22.2	
2	20.9	24.5	25.0	21.0	
3+	19.0	33.7	45.6	49.0	
CVDs and diabetes					
Heart attack: ever diagnosed/currently having	8.7	14.2	20.1	26.5	<0.0001
High blood pressure or hypertension: ever diagnosed/currently having	38.1	57.4	66.1	66.1	<0.0001
High blood cholesterol: ever diagnosed/currently having	17.4	26.3	23.9	20.2	<0.0001
Stroke: ever diagnosed/currently having	2.6	4.8	9.0	8.2	<0.0001
Diabetes or high blood sugar: ever diagnosed/currently having	10.0	19.8	21.6	15.6	<0.0001
Self-perceived health—US version (SPUUS)					
Excellent	10.9	5.3	2.7	1.6	<0.0001
Very good	22.9	15.1	7.3	3.9	
Good	38.0	41.3	30.5	24.5	
Fair	19.6	24.2	34.4	34.6	
Poor	8.1	13.7	24.5	35.4	
SPHUS-2					
Very good/excellent	33.7	20.4	10.0	5.4	<0.0001
Less than very good	65.8	79.2	89.3	94.6	
EURO-D caseness					
No	78.9	76.3	63.2	45.9	<0.0001
Yes	21.1	23.7	36.8	54.1	
Parts of EURO-D (12 symptoms)					
Depression	30.0	32.6	41.4	42.8	<0.0001
Pessimism	14.4	17.2	28.0	41.2	<0.0001
Suicidality	3.6	4.7	9.0	12.5	<0.0001
Guilt	4.6	4.0	4.0	3.5	<0.0001
Sleep	24.8	29.9	34.9	44.0	<0.0001
Interest	7.8	9.6	15.5	24.5	<0.0001
Irritability	26.4	25.3	28.4	30.4	<0.0001
Appetite	6.1	7.8	9.6	17.9	<0.0001
Fatigue	29.1	36.6	47.9	63.0	<0.0001
Concentration	13.7	18.0	28.2	42.4	<0.0001
Enjoyment	8.1	10.3	15.6	24.5	<0.0001
Tearfulness	19.6	21.0	23.9	25.3	<0.0001

* The χ^2^ test was used to compare categorical variables.

**Table 5:** Multinomial logistic regression results for maximum handgrip groups of Croatian population (over 50 years) and observed variables (activities (leisure/sport); QoL parameters (CASP/SPH) and health related indicators (EURO-D/CVDs and Diabetes).

Observed Variable	Medium		*p*-Value	Low		*p*- Value
	B	OR (% CI)		B	OR (% CI)	
Activities						
done voluntary or charity work	−0.46	0.63 (0.28–1.42)	0.267	−0.54	0.59 (0.21–1.67)	0.316
attended an educational or training course	−0.32	0.73 (0.24–2.16)	0.567	−0.16	0.86 (0.2–3.57)	0.830
gone to a sport, social or other kind of club	−0.55	0.58 (0.38–0.89)	0.013	−0.48	0.62 (0.32–1.21)	0.163
taken part in a political or community-related organization	−0.19	0.83 (0.36–1.89)	0.651	0.15	1.16 (0.32–4.21)	0.817
read books, magazines or newspapers	0.33	1.4 (0.96–2.04)	0.084	0.22	1.25 (0.77–2.04)	0.368
did word or number games (crossword puzzles/Sudoku/etc.)	0.07	1.07 (0.69–1.66)	0.772	0.02	1.02 (0.58–1.79)	0.953
played cards or games such as chess	−0.30	0.74 (0.5–1.1)	0.141	−0.58	0.56 (0.3–1.04)	0.066
No additional sport activities	0.37	1.45 (1.23–1.7)	0.000	0.66	1.94 (1.59–2.37)	0.000
Physical inactivity	0.13	1.14 (0.39–3.3)	0.807	0.53	1.7 (0.52–5.63)	0.382
Dietary intake						
Dairy products	−0.04	0.96 (0.83–1.11)	0.614	−0.01	0.99 (0.82–1.19)	0.907
Legumes and eggs	−0.15	0.86 (0.72–1.03)	0.098	−0.25	0.78 (0.62–0.97)	0.024
Meat, fish and poultry	0.13	1.14 (0.86–1.51)	0.375	0.11	1.12 (0.8–1.56)	0.509
Fruit and vegetable	0.05	1.06 (0.83–1.35)	0.660	−0.01	0.99 (0.72–1.36)	0.934
CVDs and diabetes						
Heart attack: ever diagnosed/currently having	−0.35	0.71 (0.39–1.27)	0.248	0.04	1.04 (0.51–2.15)	0.911
High blood pressure or hypertension: ever diagnosed/currently having	0.42	1.52 (0.99–2.34)	0.054	0.45	1.57 (0.92–2.69)	0.101
High blood cholesterol: ever diagnosed/currently having	−0.31	0.73 (0.43–1.23)	0.239	−0.32	0.72 (0.38–1.37)	0.323
Stroke: ever diagnosed/currently having	−0.33	0.72 (0.3–1.72)	0.459	0.28	1.32 (0.45–3.85)	0.608
Diabetes or high blood sugar: ever diagnosed/currently having	0.11	1.12 (0.65–1.94)	0.688	0.06	1.07 (0.54–2.11)	0.855
Number of chronic diseases	0.13	1.14 (0.89–1.44)	0.300	0.27	1.31 (0.99–1.73)	0.058
EURO-D						
Depression	0.04	1.04 (0.6–1.8)	0.890	0.13	1.14 (0.6–2.2)	0.686
Pessimism	0.29	1.34 (0.8–2.24)	0.271	0.29	1.34 (0.71–2.52)	0.370
Suicidality	−0.32	0.73 (0.24–2.23)	0.581	0.53	1.7 (0.49–5.89)	0.405
Guilt	−1.27	0.28 (0.12–0.68)	0.005	−1.08	0.34 (0.12–0.98)	0.045
Sleep	0.14	1.15 (0.69–1.91)	0.594	−0.12	0.89 (0.48–1.64)	0.704
Interest	0.02	1.02 (0.48–2.18)	0.959	0.05	1.05 (0.43–2.55)	0.913
Irritability	−0.33	0.72 (0.44–1.19)	0.204	−0.63	0.54 (0.29–0.99)	0.048
Appetite	−0.27	0.76 (0.34–1.72)	0.510	−0.32	0.72 (0.28–1.88)	0.508
Fatigue	−0.03	0.97 (0.61–1.54)	0.903	0.35	1.41 (0.81–2.48)	0.225
Concentration	0.20	1.22 (0.66–2.24)	0.521	0.65	1.91 (0.94–3.89)	0.073
Enjoyment	−0.12	0.88 (0.46–1.71)	0.711	0.03	1.03 (0.46–2.3)	0.934
Tearfulness	−0.31	0.73 (0.34–1.58)	0.430	−0.16	0.85 (0.37–1.98)	0.712
Euro-D (yes)	0.25	1.29 (0.54–3.06)	0.563	0.07	1.07 (0.39–2.96)	0.891
CASP, low	1.82	6.17 (0.08–481.4)	0.413	4.63	10.15 (0.71–14.58)	0.068
CASP, medium	1.18	3.26 (0.44–24.25)	0.249	2.33	10.28 (0.99–16.26)	0.051
CASP, high	1.23	3.41 (0.5–23.07)	0.209	2.30	9.99 (1.08–92.77)	0.043
Sphus1	0.20	1.22 (0.9–1.66)	0.201	0.35	1.42 (0.98–2.06)	0.061
Sphus2	−0.28	0.75 (0.39–1.46)	0.402	−0.13	0.88 (0.38–2.03)	0.757
BMI	0.60	0.55 (0.33–0.9)	0.017	0.84	0.43 (0.24–0.78)	0.005
Retired	0.01	1.01 (0.98–1.04)	0.615	0.02	1.02 (0.98–1.05)	0.367
Marital status	0.08	1.08 (0.95–1.23)	0.222	0.33	1.39 (1.21–1.6)	<0.001
Education level	−0.06	0.94 (0.88–1)	0.061	−0.09	0.91 (0.84–0.99)	0.021

CVDs: cardiovascular diseases; CASP: measure of quality of life in older age; EURO-D: depression symptoms scale used in European countries (resulting scale consists of: depression, pessimism, suicidality, guilt, sleep, interest, irritability, appetite, fatigue, concentration, enjoyment, and tearfulness).

## Data Availability

The SHARE data are distributed by SHARE-ERIC (Survey of Health, Ageing and Retirement in Europe—European Research Infrastructure Consortium) to registered users through the SHARE Research Data Center. The data used in this study were obtained from SHARE Waves 1–9, including the two waves of the SHARE Corona Survey, and are publicly available upon approval by the SHARE Research Data Center (https://share-eric.eu/data/data-access). For this study was used the https://doi.org/10.6103/SHARE.w9.900 (accessed on 14 May 2024).

## Abbreviations

The following abbreviations are used in this manuscript:

BF	Body fat
BM	Body mass
BMI	Body mass index
CVD	Cardiovascular disease
CASP	Control, Autonomy, Self-realization, and Pleasure scale
CI	Confidence interval
EURO-D	depression symptoms scale used in European countries. The resulting scale consists of: depression, pessimism, suicidality, guilt, sleep, interest, irritability, appetite, fatigue, concentration (onreading or entertainment), enjoyment, and tearfulness
OR	Odds ratio
SPH	Self-perceived Health
